# Supplementary material for: Drug repurposing for aging research using model organisms
Source: Aging Cell. 2017 Jun 16;16(5):1006–15. doi: 10.1111/acel.12626 (PMC5595691; doi:10.1111/acel.12626)
Supplement: Supplementary file 7 — Data S1 Zip‐Archive of all report cards. [file ACEL-16-1006-s007.zip › RC_PLP.pdf]

PLP

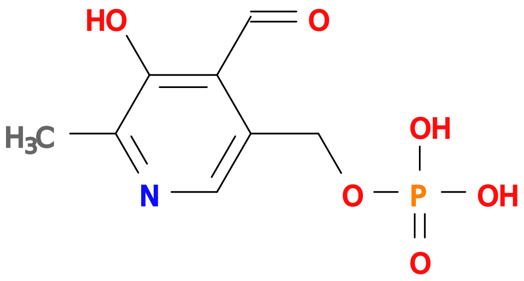

Database identifiers

ChEMBLCompound	CHEMBL82202
DrugBank	DB00114
CHEBI	18405
eMolecules	1988700

Ranking

	Rank	Score
Drosophila	170/697	0.728
C. elegans	124/591	0.302

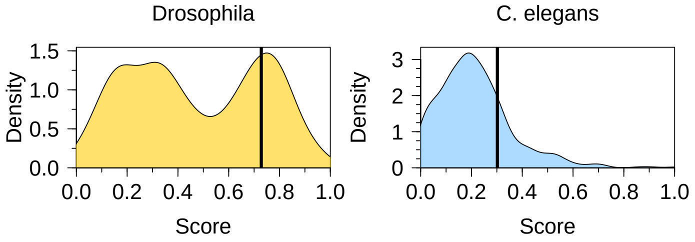

	Ageing implication	Domain conservation	Binding site conservation	Binding affinity	Bioavailability	Lipinski	Promiscuity	Purchasability	Drug approval	**Total**
Drosophila	1.0	0.982	1.0	0.653	(0.9)	0.0	-0.024	0.1	0.075	0.728
C. elegans	1.0	0.915	0.946	0.653	0.267	0.0	-0.024	0.1	0.075	0.302

Names

- **pyridoxal 5'-phosphate**
- codecarboxylase
- pyridoxal 5-monophosphoric acid ester
- pyridoxal 5'-(dihydrogen phosphate)

Roles

coenzyme, EC 2.7.7.7 (DNA-directed DNA polymerase) inhibitor, human metabolite, Escherichia coli metabolite, Saccharomyces cerevisiae metabolite

Status

Approved drug (according to ChEMBL)	No
Classification (according to DrugBank)	nutraceutical
Number of Rule of 5 violations	0
Binding affinity to original target in log units (RF-Score prediction)	5.63
Burns *C. elegans* bioavailability prediction	-1.39

Medical Information

Indicatation: For nutritional supplementation and for treating dietary shortage or imbalance.
Mechanism of action: Pyridoxal Phosphate is a coenzyme of many enzymatic reactions. It is the active form of vitamin B6 which comprises three natural organic compounds, pyridoxal, pyridoxamine and pyridoxine. Pyridoxal phosphate acts as a coenzyme in all transamination reactions, and in some decarboxylation and deamination reactions of amino acids. The aldehyde group of pyridoxal phosphate forms a Schiff-base linkage with the epsilon-amino group of a specific lysine group of the aminotransferase enzyme. The alpha-amino group of the amino acid substrate displaces the epsilon-amino group of the active-site lysine residue. The resulting aldimine becomes deprotonated to become a quinoid intermediate, which in turn accepts a proton at a different position to become a ketimine. The resulting ketimine is hydrolysed so that the amino group remains on the protein complex.
Retail: Pamlab LLC, Physicians Total Care Inc., Red River Pharma Manufacturing

(Information from DrugBank)

Compound Target Characteristics

Aromatic-L-amino-acid decarboxylase

Best gene implication in ageing for this target family came from gene P14173 annotated in UniProt release 2014_02. Annotation GO subterm of 7568 (aging) was Inferred from Expression Pattern

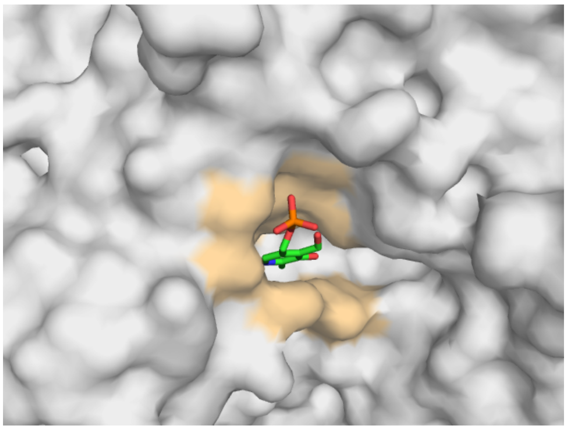

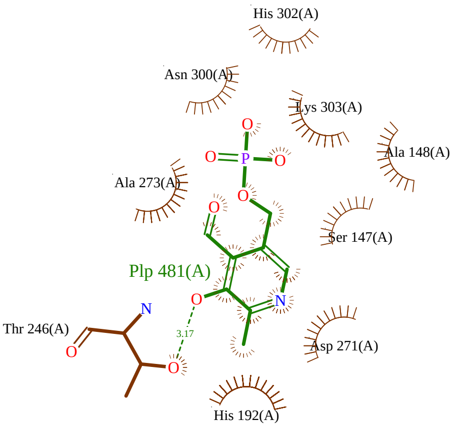

protein	amino acids contacts (binding site)
PDB:3rbf:chainA:P20711	S A S H T D A N H K
sp:P20711:DDC_HUMAN	S A S H T D A N H K
tr:D3ZMA5:D3ZMA5_RAT	S A S H T D A N H K
sp:P14173:DDC_RAT	S A S H T D A N H K
sp:O88533:DDC_MOUSE	S A S H T D A N H K
tr:Q5SUV8:Q5SUV8_MOUSE	S A S H T D A N H K
tr:F0JAJ7:F0JAJ7_DROME	T A S H T D A N H K
tr:Q9VIZ9:Q9VIZ9_DROME	S A S N T D A N H K
tr:C9QP35:C9QP35_DROME	S A S N T D A N H K
tr:H5V873:H5V873_DROME	S A S N T D A N H K
sp:P05031:DDC_DROME	T A S H T D A N H K
sp:P18486:L2AM_DROME	S A S N T D A N H K
tr:Q5BID9:Q5BID9_DROME	S A S N T D A N H K
tr:F0JAJ6:F0JAJ6_DROME	T A S H T D A N H K
tr:Q66NI4:Q66NI4_CAEEL	T A S H T D A N H K
tr:Q66NI3:Q66NI3_CAEEL	T A S H T D A N H K
tr:H2KYG4:H2KYG4_CAEEL	T A S H T D A N H K
tr:O45137:O45137_CAEEL	T A S H T D A N H K

	whole protein		domain-based		contact-based	
protein	ident	simil	ident	simil	ident	simil
PDB:3rbf:chainA:P20711	1.0	1.0	1.0	1.0	1.0	1.0
sp:P20711:DDC_HUMAN	1.0	1.0	1.0	1.0	1.0	1.0
tr:D3ZMA5:D3ZMA5_RAT	0.81	0.89	0.78	0.84	1.0	1.0
sp:P14173:DDC_RAT	0.89	0.97	0.91	0.98	1.0	1.0
sp:O88533:DDC_MOUSE	0.89	0.97	0.92	0.98	1.0	1.0
tr:Q5SUV8:Q5SUV8_MOUSE	0.89	0.97	0.92	0.98	1.0	1.0
tr:F0JAJ7:F0JAJ7_DROME	0.6	0.87	0.66	0.88	0.9	0.95
tr:Q9VIZ9:Q9VIZ9_DROME	0.42	0.77	0.5	0.84	0.9	0.94
tr:C9QP35:C9QP35_DROME	0.44	0.78	0.5	0.84	0.9	0.94
tr:H5V873:H5V873_DROME	0.44	0.78	0.5	0.84	0.9	0.94
sp:P05031:DDC_DROME	0.56	0.81	0.66	0.88	0.9	0.95
sp:P18486:L2AM_DROME	0.44	0.78	0.5	0.84	0.9	0.94
tr:Q5BID9:Q5BID9_DROME	0.42	0.77	0.5	0.83	0.9	0.94
tr:F0JAJ6:F0JAJ6_DROME	0.56	0.81	0.66	0.88	0.9	0.95
tr:Q66NI4:Q66NI4_CAEEL	0.38	0.7	0.4	0.73	0.9	0.95
tr:Q66NI3:Q66NI3_CAEEL	0.38	0.69	0.39	0.71	0.9	0.95
tr:H2KYG4:H2KYG4_CAEEL	0.4	0.75	0.4	0.73	0.9	0.95
tr:O45137:O45137_CAEEL	0.39	0.74	0.39	0.71	0.9	0.95

Ddc-RB (UniProt:F0JAJ7) annotation
Cofactor: pyridoxal 5'-phosphate Evidence=(RuleBase:RU000382);

(Information from UniProt)

amd (FBgn0000075) associated phenotypes
chemical sensitive, dominant, partially

(Information from FlyBase)

amd (UniProt:Q9VIZ9) annotation
Cofactor: pyridoxal 5'-phosphate Evidence=(RuleBase:RU000382);

(Information from UniProt)

amd-RA (UniProt:C9QP35) annotation
Cofactor: pyridoxal 5'-phosphate Evidence=(RuleBase:RU000382);

(Information from UniProt)

amd-RA (UniProt:H5V873) annotation
Cofactor: pyridoxal 5'-phosphate Evidence=(RuleBase:RU000382);

(Information from UniProt)

Ddc (FBgn0000422) associated phenotypes
developmental rate defective, flight defective, heat sensitive, learning defective, lethal - all die before end of prepupal stage, lethal - all die before end of pupal stage, locomotor behavior defective, memory defective, partially, small body, somatic clone, some die during prepupal stage, some die during pupal stage, some die during third instar larval stage, wound healing defective

(Information from FlyBase)

Ddc (UniProt:P05031) annotation
Function: Catalyzes the decarboxylation of L-3,4- dihydroxyphenylalanine (DOPA) to dopamine, L-5-hydroxytryptophan to serotonin and L-tryptophan to tryptamine. Variation in the synthesis of bioamines may be a factor contributing to natural variation in life span. (PubMed:12881721).
Cofactor: pyridoxal 5'-phosphate
Subunit: Homodimer.
Tissue specificity: Hypoderm isoform is expressed only in hypodermal epithelium and the CNS isoform only in central nervous system. (PubMed:3095924, PubMed:3102230).
Developmental stage: Hypoderm isoform has high expression levels in hypoderm during late embryogenesis, late larval development, pupariation and adult eclosion. CNS isoform has constant expression level in CNS throughout the life cycle. (PubMed:3007242, PubMed:3095924).
Induction: By ecdysone. In larval epidermis, expression is rapidly induced. In adult epidermis expression responds to a pulse of hormone and there is a time lag between initial exposure and appearance of DDC. (PubMed:3007239).
Polymorphism: Three common molecular polymorphisms (2 in the promoter region and Phe-12) account for 15.5 (Information from UniProt)

amd (UniProt:P18486) annotation
Function: Participates in catecholamine metabolism. It plays a vital role in cuticle development.
Cofactor: pyridoxal 5'-phosphate Evidence=;
Developmental stage: Reaches a maximum in mid-embryogenesis.

(Information from UniProt)

amd (UniProt:Q5BID9) annotation
Cofactor: pyridoxal 5'-phosphate Evidence=(RuleBase:RU000382);

(Information from UniProt)

Ddc-RC (UniProt:F0JAJ6) annotation
Cofactor: pyridoxal 5'-phosphate Evidence=(RuleBase:RU000382);

(Information from UniProt)

bas-1 (UniProt:Q66NI4) annotation
Cofactor: pyridoxal 5'-phosphate Evidence=(RuleBase:RU000382);

(Information from UniProt)

bas-1 (UniProt:Q66NI3) annotation
Cofactor: pyridoxal 5'-phosphate Evidence=(RuleBase:RU000382);

(Information from UniProt)

bas-1 (WBGene00000239) associated phenotypes
antibody staining variant, aqueous adaptation defective, dopamine deficient, male mating variant, male turning defective, serotonin deficient

(Information from WormBase)

bas-1 (UniProt:H2KYG4) annotation
Cofactor: pyridoxal 5'-phosphate Evidence=(RuleBase:RU000382);

(Information from UniProt)

bas-1 (UniProt:O45137) annotation
Cofactor: pyridoxal 5'-phosphate Evidence=(RuleBase:RU000382);

(Information from UniProt)

Cystathionine beta-synthase

Best gene implication in ageing for this target family came from gene Q9VRD9 annotated in UniProt release 2014_02. Annotation GO 8340 (determination of adult lifespan) was Inferred from Mutant Phenotype

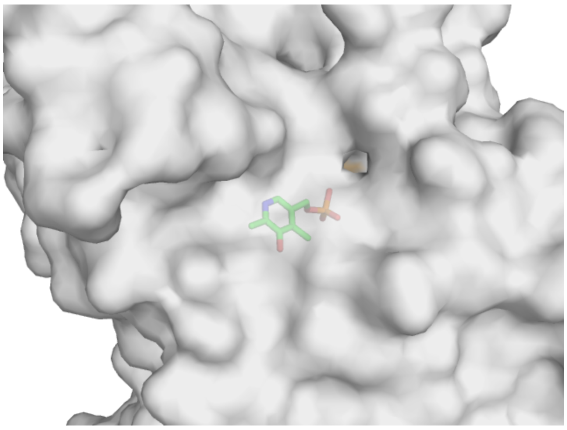

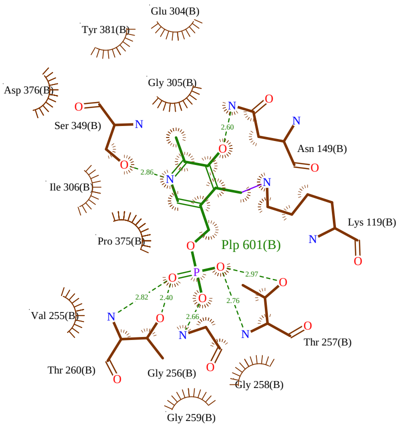

protein	amino acids contacts (binding site)																	
PDB:1jbq:chainA:P35520	K	S	N	S	V	G	T	G	G	T	E	G	I	S	P	D	Y
PDB:1m54:chainA:P35520	K	S	N	S	V	G	T	G	G	T	E	G	I	S	P	D	Y
PDB:4coo:chainA:P35520	K	S	N	S	V	G	T	G	G	T	E	G	I	S	P	D	Y
PDB:4l0d:chainA:P35520	K	S	N	S	V	G	T	G	G	T	E	G	I	S	P	D	Y
PDB:4l27:chainB:P35520	K	S	N	S	V	G	T	G	G	T	E	G	I	S	P	D	Y
PDB:4l28:chainA:P35520	K	S	N	S	V	G	T	G	G	T	E	G	I	S	P	D	Y
PDB:4l3v:chainB:P35520	K	S	N	S	V	G	T	G	G	T	E	G	I	S	P	D	Y
tr:F5H2U1:F5H2U1_HUMAN	K	S	N	S	V	G	T	G	G	T	E	G	I	S	P	D	Y
sp:P35520:CBS_HUMAN	K	S	N	S	V	G	T	G	G	T	E	G	I	S	P	D	Y
sp:P32232:CBS_RAT	K	S	N	S	A	G	T	G	G	T	E	G	I	S	P	D	Y
sp:Q91WT9:CBS_MOUSE	K	S	N	S	A	G	T	G	G	T	E	G	I	S	P	D	Y
tr:Q9VRD9:Q9VRD9_DROME	K	S	N	S	A	G	T	A	G	T	E	G	I	S	P	D	Y
tr:Q9N4K2:Q9N4K2_CAEEL	K	S	N	G	A	G	T	G	G	T	E	G	I	S	P	D	Y
tr:G5EFH8:G5EFH8_CAEEL	K	S	N	T	A	G	T	G	G	T	E	G	I	S	P	D	Y
sp:P32582:CBS_YEAST	K	S	N	G	A	G	T	G	G	T	E	G	I	S	P	D	Y
tr:E9P980:E9P980_YEASX	K	S	N	G	A	G	T	G	G	T	E	G	I	S	P	D	Y

protein	whole protein ident	simil	domain-based ident	simil	contact-based ident	simil
PDB:1jbq:chainA:P35520	1.0	1.0	1.0	1.0	1.0	1.0
PDB:1m54:chainA:P35520	1.0	1.0	1.0	1.0	1.0	1.0
PDB:4coo:chainA:P35520	1.0	1.0	1.0	1.0	1.0	1.0
PDB:4l0d:chainA:P35520	1.0	1.0	1.0	1.0	1.0	1.0
PDB:4l27:chainB:P35520	1.0	1.0	1.0	1.0	1.0	1.0
PDB:4l28:chainA:P35520	1.0	1.0	1.0	1.0	1.0	1.0
PDB:4l3v:chainB:P35520	1.0	1.0	1.0	1.0	1.0	1.0
tr:F5H2U1:F5H2U1_HUMAN	0.84	0.84	0.97	0.97	1.0	1.0
sp:P35520:CBS_HUMAN	1.0	1.0	1.0	1.0	1.0	1.0
sp:P32232:CBS_RAT	0.82	0.92	0.91	0.97	0.94	0.96
sp:Q91WT9:CBS_MOUSE	0.82	0.92	0.93	0.98	0.94	0.96
tr:Q9VRD9:Q9VRD9_DROME	0.47	0.76	0.65	0.89	0.88	0.93
tr:Q9N4K2:Q9N4K2_CAEEL	0.24	0.39	0.56	0.84	0.88	0.93
tr:G5EFH8:G5EFH8_CAEEL	0.22	0.37	0.55	0.83	0.88	0.93
sp:P32582:CBS_YEAST	0.35	0.64	0.53	0.83	0.88	0.93
tr:E9P980:E9P980_YEASX	0.35	0.64	0.53	0.83	0.88	0.93

Cbs (FBgn0031148) associated phenotypes

RU486 conditional, long lived, short lived

(Information from FlyBase)

Cbs (UniProt:Q9VRD9) annotation

Cofactor: pyridoxal 5'-phosphate Evidence=(RuleBase:RU361204);

(Information from UniProt)

Serine racemase

Best gene implication in ageing for this target family came from gene Q76EQ0 annotated in UniProt release 2014_02. Annotation GO 7568 (aging) was Inferred from Expression Pattern

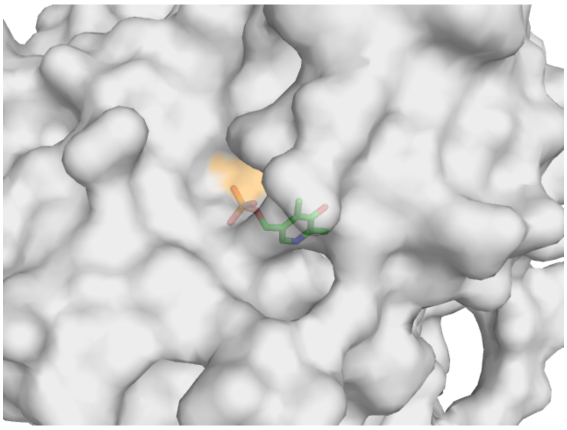

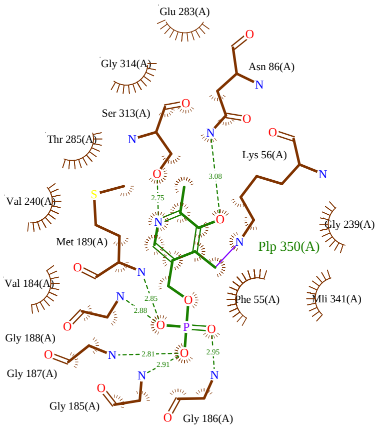

protein	amino acids contacts (binding site)
PDB:3hmk:chainA:Q76EQ0	F K N N V G G G G M G V E T S G
PDB:3l6c:chainA:Q76EQ0	F K N N V G G G G M G V E T S G
tr:Q8N3F4:Q8N3F4_HUMAN	F K N N V G G G G M G V E T S G
sp:Q9GZT4:SRR_HUMAN	F K N N V G G G G M G V E T S G
sp:Q76EQ0:SRR_RAT	F K N N V G G G G M G V E T S G
sp:Q9QZX7:SRR_MOUSE	F K N N V G G G G M G V E T S G
tr:Q93968:Q93968_CAEEL	F K N Y V G G G G L G V E T C G
sp:P36007:SRY1_YEAST	F K N Y L G G G G L G A E T S G

protein	whole protein ident	simil	domain-based ident	simil	contact-based ident	simil
PDB:3hmk:chainA:Q76EQ0	1.0	1.0	1.0	1.0	1.0	1.0
PDB:3l6c:chainA:Q76EQ0	0.99	1.0	1.0	1.0	1.0	1.0
tr:Q8N3F4:Q8N3F4_HUMAN	0.76	0.81	0.83	0.89	1.0	1.0
sp:Q9GZT4:SRR_HUMAN	0.89	0.96	0.92	0.98	1.0	1.0
sp:Q76EQ0:SRR_RAT	1.0	1.0	1.0	1.0	1.0	1.0
sp:Q9QZX7:SRR_MOUSE	0.94	0.97	0.96	0.99	1.0	1.0
tr:Q93968:Q93968_CAEEL	0.35	0.71	0.38	0.74	0.81	0.84
sp:P36007:SRY1_YEAST	0.39	0.77	0.42	0.79	0.75	0.85

Serine racemase

Best gene implication in ageing for this target family came from gene Q76EQ0 annotated in UniProt release 2014_02. Annotation GO 7568 (aging) was Inferred from Expression Pattern

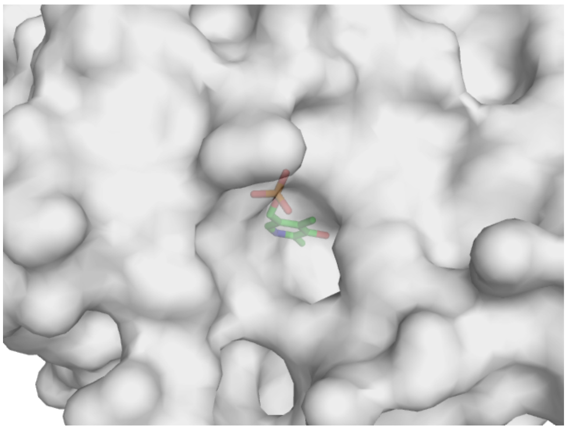

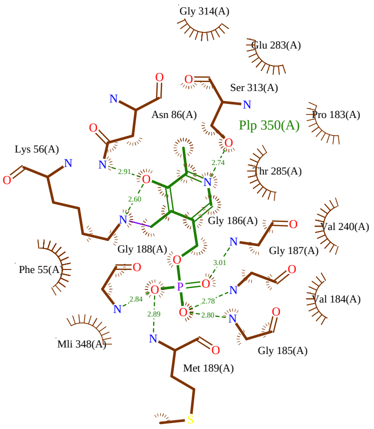

protein	amino acids contacts (binding site)														
PDB:3l6b:chainA:Q9GZT4	F	K	N	P	V	G	G	G	G	M	V	E	T	S	G
tr:Q8N3F4:Q8N3F4_HUMAN	F	K	N	P	V	G	G	G	G	M	V	E	T	S	G
sp:Q9GZT4:SRR_HUMAN	F	K	N	P	V	G	G	G	G	M	V	E	T	S	G
sp:Q76EQ0:SRR_RAT	F	K	N	P	V	G	G	G	G	M	V	E	T	S	G
sp:Q9QZX7:SRR_MOUSE	F	K	N	P	V	G	G	G	G	M	V	E	T	S	G
tr:Q93968:Q93968_CAEEL	F	K	N	S	V	G	G	G	G	L	V	E	T	C	G
sp:P36007:SRY1_YEAST	F	K	N	P	L	G	G	G	G	L	A	E	T	S	G

protein	whole protein ident	simil	domain-based ident	simil	contact-based ident	simil
PDB:3l6b:chainA:Q9GZT4	1.0	1.0	1.0	1.0	1.0	1.0
tr:Q8N3F4:Q8N3F4_HUMAN	0.85	0.85	0.91	0.91	1.0	1.0
sp:Q9GZT4:SRR_HUMAN	1.0	1.0	1.0	1.0	1.0	1.0
sp:Q76EQ0:SRR_RAT	0.89	0.96	0.91	0.98	1.0	1.0
sp:Q9QZX7:SRR_MOUSE	0.9	0.97	0.9	0.97	1.0	1.0
tr:Q93968:Q93968_CAEEL	0.36	0.7	0.38	0.73	0.8	0.87
sp:P36007:SRY1_YEAST	0.39	0.76	0.42	0.78	0.8	0.93

Cystathionine beta-synthase

Best gene implication in ageing for this target family came from gene Q9VRD9 annotated in UniProt release 2014_02. Annotation GO 8340 (determination of adult lifespan) was Inferred from Mutant Phenotype

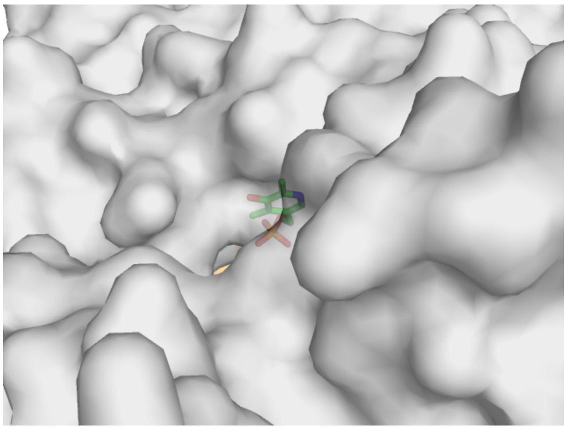

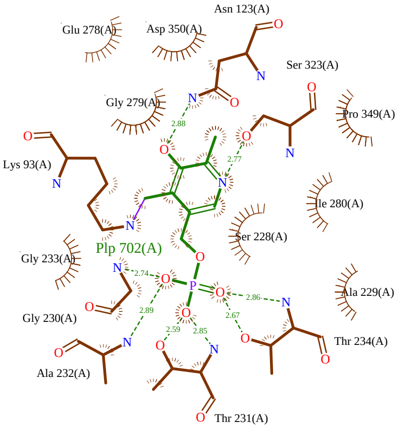

protein	amino acids contacts (binding site)														
PDB:3pc2:chainA:Q9VRD9	K	N	S	A	G	T	A	G	T	E	G	I	S	P	D
tr:F5H2U1:F5H2U1_HUMAN	K	N	S	V	G	T	G	G	T	E	G	I	S	P	D
sp:P35520:CBS_HUMAN	K	N	S	V	G	T	G	G	T	E	G	I	S	P	D
sp:P32232:CBS_RAT	K	N	S	A	G	T	G	G	T	E	G	I	S	P	D
sp:Q91WT9:CBS_MOUSE	K	N	S	A	G	T	G	G	T	E	G	I	S	P	D
tr:Q9VRD9:Q9VRD9_DROME	K	N	S	A	G	T	A	G	T	E	G	I	S	P	D
tr:Q9N4K2:Q9N4K2_CAEEL	K	N	G	A	G	T	G	G	T	E	G	I	S	P	D
tr:G5EFH8:G5EFH8_CAEEL	K	N	T	A	G	T	G	G	T	E	G	I	S	P	D
sp:P32582:CBS_YEAST	K	N	G	A	G	T	G	G	T	E	G	I	S	P	D
tr:E9P980:E9P980_YEASX	K	N	G	A	G	T	G	G	T	E	G	I	S	P	D

	whole protein		domain-based		contact-based	
protein	ident	simil	ident	simil	ident	simil
PDB:3pc2:chainA:Q9VRD9	1.0	1.0	1.0	1.0	1.0	1.0
tr:F5H2U1:F5H2U1_HUMAN	0.45	0.72	0.64	0.87	0.87	0.92
sp:P35520:CBS_HUMAN	0.47	0.77	0.65	0.89	0.87	0.92
sp:P32232:CBS_RAT	0.46	0.74	0.67	0.89	0.93	0.96
sp:Q91WT9:CBS_MOUSE	0.46	0.74	0.67	0.89	0.93	0.96
tr:Q9VRD9:Q9VRD9_DROME	1.0	1.0	1.0	1.0	1.0	1.0
tr:Q9N4K2:Q9N4K2_CAEEL	0.23	0.37	0.56	0.83	0.87	0.92
tr:G5EFH8:G5EFH8_CAEEL	0.22	0.35	0.57	0.84	0.87	0.92
sp:P32582:CBS_YEAST	0.37	0.66	0.54	0.82	0.87	0.92
tr:E9P980:E9P980_YEASX	0.36	0.66	0.53	0.82	0.87	0.92

Cbs (FBgn0031148) associated phenotypes

RU486 conditional, long lived, short lived

(Information from FlyBase)

Cbs (UniProt:Q9VRD9) annotation

Cofactor: pyridoxal 5'-phosphate Evidence=(RuleBase:RU361204);

(Information from UniProt)
